# The experimental demonstration of a topological current divider

**DOI:** 10.1038/s41467-023-39503-4

**Published:** 2023-06-22

**Authors:** Francesco Romeo, Antonio Di Bartolomeo

**Affiliations:** 1 Dipartimento di Fisica “E. R. Caianiello”, Università di Salerno, I-84084 Fisciano, Italy; 2 INFN, Sez. di Napoli, Gruppo Collegato di Salerno, I-84084 Fisciano, Italy

**Keywords:** Electronic devices, Topological insulators

## Abstract

Topological electronics is an emerging field aiming at exploiting the topological properties of matter in devices with extended functionalities. Recent experiments demonstrate a topological current divider, a key circuit element for this emerging technology.

The generation and control of charge current is the basis for conventional *electronics*, which has had a huge societal impact. Recently, alternative paradigms are the subject of intense investigations. For instance, *spintronics*^1^ and *spin-orbitronics*^2^ are oriented towards the use of electron spin and angular momentum to implement devices with new working principles. While these technologies are based on intrinsic properties of the electrons (i.e., charge, spin, angular momentum), new opportunities are emerging from the non-trivial band structure properties of new nanostructured materials. *Valleytronics*^3^, one of these options, exploits the local minima (i.e. the so-called valleys) of the band structure as an effective degree of freedom. Lately, the groundbreaking concept of *topotronics*^4^ (topological electronics) is leading to a further paradigm shift. Topological electronics aims at obtaining devices exploiting the topological properties (in mathematical sense) of the material band structure. Topological superconductors and insulators are important members of the quantum matter family of interest in *topotronics*. While the superconductors require low temperatures, topological insulators, at least in principle, have the potential to be the building blocks of topological devices working at higher temperatures. Thus, identifying materials, dimensionality, device layout and functionalities represents a critical step to fully exploit the exotic properties of quantum materials.

## Topological quantum materials for high-temperature devices

In the last decade, quantum matter^5^ with topological properties has aroused great interest within the condensed matter community. This family includes two-dimensional topological insulators^6^, considered promising candidates to implement high-temperature topological circuits and nanodevices with new properties and functionalities. In essence, a two-dimensional topological insulator is a bidimensional material exhibiting features of an ordinary electrical insulator in the bulk, while presenting helical one-dimensional edge modes, originating an electrical conduction protected by the existence of topological invariants of the electronic bands (e.g. the so-called Chern numbers). Edge modes behave like perfect one-dimensional conductors, which are very appealing in view of their potential in *spintronics*. The band structure is characterized by a bulk gap accompanied by in-gap states corresponding to the edge modes and presenting exotic properties. The bulk gap is an important figure of merit; if it exceeds the room-temperature thermal energy (i.e. $\approx 25{meV}$), as it often occurs in topological insulators^7^, then topological protection survives at high temperature and, possibly, up to room temperature. Moreover, bidimensionality represents a desirable feature in view of easier integration in conventional industrial technologies.

Recently, based on the extraordinary properties of two-dimensional topological insulators, Bing-Lan Wu and coworkers^8^ have theoretically proposed a device layout that implements programmable integrated circuits by using disordered Chern insulators. These authors have demonstrated that gate-induced step voltages can be efficiently used to obtain spatially adjustable channels, which are robust against backscattering events, gate voltage fluctuations and disorder strength. The main conclusion of Ref. ^8^ is that interface topological channels with arbitrary and programmable trajectories can be constructed and controlled by external gates. The study has profound implications because logic gates with potential applications in low power-integrated circuits can be obtained. These programmable circuits, based on the controllable coexistence of Chern domains, also realize the partition of the current in branch wires whose number can be adjusted by using external gates.

The current partition in topological systems is non-trivial so that the implementation of a topological current divider^9^ represents an important achievement.

Writing in *Nature Communications*, Dmitry Ovchinnikov et al.^10^ reported the realization of a topological platform inspired by the theoretical proposal by Bing-Lan Wu and coworkers^8^ and the implementation of a topological current divider.

## A current divider and its topological counterpart

In conventional electronics, a current divider can be implemented by a simple linear circuit (e.g. the parallel connection of two resistors biased by a current) able to split an input current $I$ into two branches whose currents are denoted by ${I}_{a}$ and ${I}_{b}$, respectively. Charge conservation implies that $I={I}_{a}+{I}_{b}$, while the intensity of each branch current is a fraction of the input current determined solely by the branch electrical resistances ${R}_{a}$ and ${R}_{b}$, so that ${I}_{a}/{I}_{b}={R}_{b}/{R}_{a}$.

Conversely, in a topological current divider, the relative intensity of the branch currents reflects the topological properties of the material and it is not (uniquely) affected by the branch electrical resistances.

## Chern domains for programmable topological electronics

**Fig. 1:**
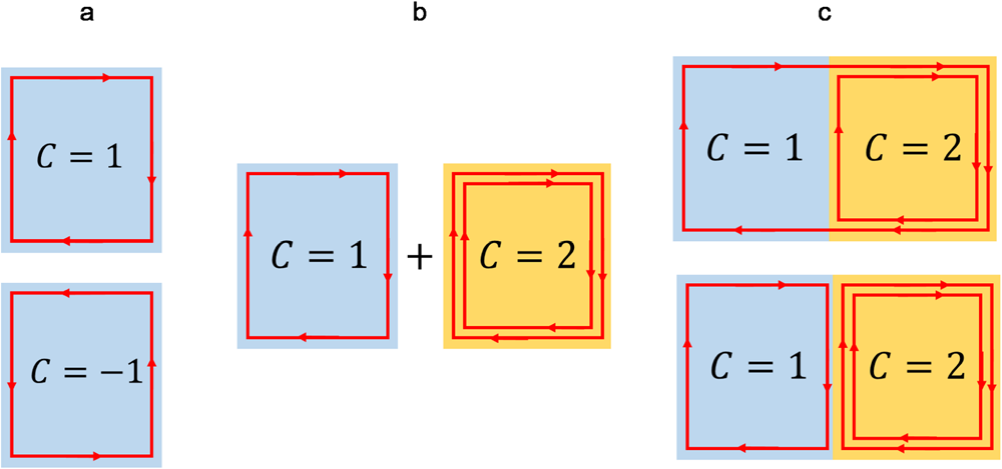
Formation of a Chern junction. **a** Chern insulators with $C=1$ corresponding to the clockwise chirality and $C=-1$ corresponding to the counterclockwise chirality. The edge mode chirality is controlled by an external magnetic field. **b** Chirality depends on the material thickness. A thinner flake hosts a chiral edge mode with $C=1$, while a thicker flake presents two chiral edge modes with ${C}=2$. A Chern junction is formed in flakes with an appropriate step in thickness. **c** The picture shows schematically two of the possible scenarios for the chiral edge states at the junction.

In their experiments, Dmitry Ovchinnikov et al. succeeded in intentionally creating a “Chern junction” in a van der Waals topological antiferromagnet (MnBi_2_Te_4_), in the form of a flake with a layered structure. In few-layer samples, an exotic phase, the Chern insulator state^11^, can be stabilized by means of external magnetic fields, electrostatic gating and thickness control. These experimental knobs enable the precise manipulation of the magnetic state, the chemical potential and the topological nature of the sample. As a result, a Chern insulator, i.e. a two-dimensional magnetic topological insulator, is obtained. The latter exhibits one-dimensional chiral edge states, in which electrons travel strictly in only one direction because backscattering phenomena are topologically forbidden. In view of the special interplay between topology and time reversal symmetry breaking, edge states propagate along a direction determined by the sample magnetization (Fig. 1a). The number of chiral edge modes is equal to the Chern number $C$ which determines observable effects such as a quantized Hall resistance ${R}_{{xy}}\,={h}/\left|C\right|{e}^{2}$, where $h$ is the Planck’s constant and $e$ is the elementary charge.

Dmitry Ovchinnikov et al. observed that the Chern number $C$, defining the topological properties of the material, is controlled by the number of layers of the sample. In particular, the $C=1$ state appears in flakes with thickness corresponding to 4 and 5 layers, while the recently identified $C=2$ state is obtained in flakes with more than 6 layers. Thus, the thinner and thicker parts of a single flake containing a thickness discontinuity (routinely occurring in the fabrication process of two-dimensional layered materials^12^) can be tuned to simultaneously host topological domains with $C=1$ and $C=2$, respectively (Fig. 1b, c).

**Fig. 2:**
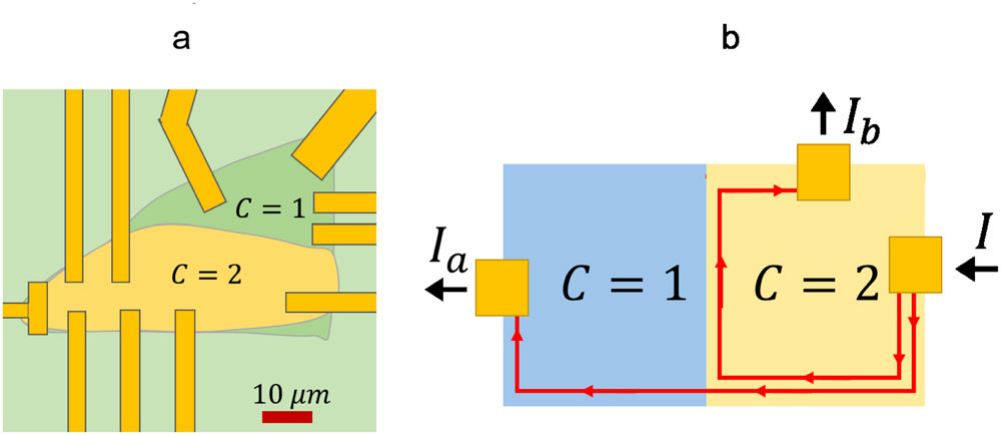
Harnessing Chern domains for topological circuits. **a** A topological current divider can be obtained by using multiterminal technology. **b** The input current $I$ is asymmetrically partitioned in edge currents ${I}_{a}$ and ${I}_{b}$, with $I={I}_{a}+{I}_{b}$. An external magnetic field and the back gate control determine the partition ratio ${I}_{a}/{I}_{b}$. The current paths and the device operation can be controlled by changing the magnetic field orientation.

This observation opens the door to the creation of topological heterostructures in which domains with different Chern numbers can coexist. At the boundary of such domains, a Chern insulator junction forms, whose exotic conduction properties can be probed by using multiterminal devices (Fig. 2a). By mastering these concepts and techniques, Dmitry Ovchinnikov et al. demonstrated the feasibility of topological circuits in which the chiral edge current (Fig. 1c) can be split, rerouted, or switched off by controlling the Chern numbers of the individual domains.

Measurements demonstrate that the Chern junction can function as a simple device in which a current signal can be either split between two outputs or routed to a single output by flipping the propagation direction of the chiral edge modes. The operation conditions of the device depend on the orientation of a rather intense external magnetic field (controlling the chirality of the edge modes) and on the back gate voltage.

The surprising aspect is that a topological current divider regime exists and can be tested up to relatively high temperatures ($T\, \approx \, 30{K}$), suggesting that a high-temperature topological electronics is within reach. In a typical experiment, a current $I$ coming from the region with $C=2$ is (asymmetrically) partitioned in two currents, one of which, let say ${I}_{a}$, crosses the junction, while the other, ${I}_{b}$, flows along the boundary formed between domains with distinct $C$ values (Fig. 2b).

## Recent developments and outlook

Dmitry Ovchinnikov and his team have demonstrated the feasibility of programmable topological circuits that rely on the controlled coexistence of Chern domains. This technology, which is based on the manipulation of the topological properties of materials, appears to be scalable thanks to recent advances in molecular beam epitaxy and other fabrication techniques. By allowing for precise local control of the Chern numbers, multiterminal/gate topological devices can be developed. Yi-Fan Zhao^13^ and colleagues have recently achieved significant progress in this area by successfully growing magnetic topological insulator multilayers using molecular beam epitaxy, and fabricating multiterminal devices based on Chern domains that can be controlled by small magnetic fields.

Abdulhakim Bake et al.^14^ have discussed another promising technique that involves ion-beam amorphization to create trivial insulator regions on the surface of a Sb_2_Te_3_ single crystal topological insulator. The ion-beam treatment implements an inverse lithographic process, which is reminiscent of the original theoretical proposal by Bing-Lan Wu and coworkers^8^. The technique is promising for industry applications because it has the potential of producing cm^2^ devices using fabrication processes that are compatible with CMOS technology.

These developments suggest that high-temperature topological electronics^8,15^ is going to experience a rapid growth. The recent demonstration of topological current dividers, using distinct platforms, represents a significant milestone for the implementation of an all-electrical Chern domains technology. The scientific and technological development of such technology could represent one of the most intriguing challenges that scientists and engineers will face in years to come.
